# Preparation and Characterization of Egg White Protein-Based Composite Edible Coating Containing Thymol Nanoemulsion

**DOI:** 10.3390/foods13233809

**Published:** 2024-11-26

**Authors:** Huajiang Zhang, Afeng Wei, Rui Chuang, Lina Xu, Cuiping Han, Hanyu Li, Ning Xia

**Affiliations:** College of Food Science, Northeast Agricultural University, Harbin 150030, China; hjthzhang@163.com (H.Z.); weiafeng2000@163.com (A.W.); ruichuang2004@163.com (R.C.); xulina0827@163.com (L.X.); lihanyu1004@126.com (H.L.); xianing1981@126.com (N.X.)

**Keywords:** thymol nanoemulsion, egg white protein, edible coating, antibacterial packaging

## Abstract

In this study, thymol-loaded nanoemulsion (THYNE) was incorporated into a mixture of egg white protein and hyaluronic acid to prepare antibacterial biopolymer coatings. The oil phase of the nanoemulsion (NE) was prepared by mixing different mass ratios of thymol and corn oil. NE was formed using ultrasonic emulsification, and the physicochemical properties of the NE were investigated. When the content of thymol in the oil phase was 30%, the particle size reached a minimum of 107.93 nm, PDI was 0.167, and Zeta potential was −18.2 mV, and it remained kinetically stable after 4 weeks of storage at 4 °C. Based on this study, composite coatings containing 5%, 10% and 20% THYNE were prepared, and the rheological properties, microstructure, FTIR, release properties and antibacterial properties of the coatings were investigated. The results show that the coating solutions exhibited shear thinning behavior. With increasing THYNE content, the coating structure became loose and inhomogeneous. The release rate of THY in the coatings was greater in 95% ethanol–water solution than in deionized water. In addition, the coating solutions showed stronger antibacterial activity against *Staphylococcus aureus* than against *Escherichia coli*. The egg white protein-based composite coating containing THYNE developed in this study is expected to be an antibacterial material for food packaging with sustained release performance.

## 1. Introduction

Essential oils are volatile aromatic substances, mainly secondary metabolites produced by aromatic plants, with good antibacterial activity, safety, and biodegradability. They can be used as a natural antibacterial agent for food preservation and have received widespread attention [1]. Thymol (THY) is a natural essential oil, a phenolic and hydrophobic compound that binds to bacterial proteins and breaks down and penetrates cell membranes, making it a powerful broad-spectrum antibacterial agent. It is also approved by the U.S. Food and Drug Administration (FDA) as a generally recognized as safe (GRAS) food additive and is widely used in food, pharmaceutical, and cosmetic applications [2]. This natural antimicrobial property of thymol has led to its use as an active packaging agent in edible films or coatings to extend the shelf life of food products. However, thymol has some drawbacks, such as high volatility, low water solubility, and strong odor, and its antibacterial activity decreases over time, leading to its limitations in practical applications [3]. To overcome this drawback and to improve the stability and bioavailability of THY, it can be added to food packaging materials in various forms. THY can be encapsulated in a delivery system for slow release and long-lasting antibacterial effects [4]. The commonly used methods for encapsulating thymol include nanoemulsions, Pickering emulsions, nanocapsules, microcapsules, and electrostatic spinning techniques [5]. Li et al. [6] prepared a chitosan-based Pickering emulsion loaded with thyme essential oil (TEO), which was successfully applied to strawberries in post-harvest storage. Khubaib et al. [7] successfully encapsulated thymol in nanofibers by electrostatic spinning technique, which improved the stability of thymol. Karimi-Khorrami et al. [8] prepared nanostructured lipid carriers (NLC) and NE loaded with thymol and added to alginate solution to prepare antimicrobial biopolymer films with slow-release properties. The above illustrates that the stability and antimicrobial properties of THY can be improved based on different encapsulation systems. The method of preparing NE is simple, meaning that it can improve the stability and biological activity of thymol and promote the uniform dispersion of thymol in matrix material [9].

Nanoemulsion (NE) is a nanoscale encapsulation system consisting of two immiscible phases and which is kinetically stable, with an average particle size of 20–200 nm. Compared with conventional emulsions, NE has the advantages of small droplet size, good water solubility, and its homogeneity and stability as a system, one which can efficiently encapsulate bioactive compounds for long-term storage [10]. NE preparation methods are classified into high-energy and low-energy methods. High-energy methods mainly include high-pressure homogenization, high-pressure microjet, ultrasonication, etc., while low-energy methods mainly include phase-transition temperature, phase-transition component, and spontaneous emulsification [11]. Recently, some researchers have reported the application of nanoemulsion-based encapsulation of thymol in food preservation, so as to improve its stability and antibacterial activity. Zhang et al. [12] prepared complex films containing TEO crude emulsion (TEOC) and nanoemulsion (TEON) using ultrasonication, finding that TEON-containing films had stronger antimicrobial activity than TEOC-containing films. Liu et al. [13] prepared THYNE by the co-emulsification of natural emulsifiers. The gelatin films with THYNE had a slow release and bacteriostatic effects. Robledo et al. [14] prepared THYNE using spontaneous emulsification, ultrasonication, and a combination of both methods. Quinoa protein/chitosan films containing THYNE significantly inhibited fungal growth. The above indicates that NEs prepared by different methods can be successfully used in edible films or coating matrices. Currently, ultrasonic homogenization technology is of interest for the preparation of NE due to its low production cost, easy-to-operate system, high energy efficiency, and low instrumentation requirements [15]. Ultrasonic homogenization is based on the cavitation effect of ultrasound, which is used to generate the high temperature, high pressure, strong shear, and mechanical forces needed to rupture the droplets. Emulsions produced by ultrasound have small droplet sizes and a low dispersion index and exhibit better stability and antimicrobial activity due to the increase in the active specific surface area [16]. This method has been used in several studies to produce NE.

Edible coatings are microlayers of polysaccharides, proteins, lipids, bioactive compounds, etc., which can be used individually or in combination and applied directly to the surface of the food product by dipping or spraying [17]. Fruits and vegetables are highly perishable and have high post-harvest losses, so we sought to develop a more suitable preservation coating for fruit and vegetable products based on nanoemulsions [2]. However, coatings consisting of a single biopolymer perform poorly and are not sufficient to achieve a preservation effect, as a result there is a need to compound multiple biopolymers and add other components to overcome the limitations of a single biopolymer. Considering its environmental benefits, essential oils are the most commonly used bioactive components in the preparation of edible coatings with NE. Egg white protein and hyaluronic acid are natural biopolymers with good biocompatibility, water-holding, and film-forming properties and which can be used to prepare food preservation coatings. EWP is a unique film-forming matrix and functional ingredient from a wide range of sources at low prices. HA is a large molecule linear mucopolysaccharide that is highly malleable in water and abundant in production. It is widely used as a new food ingredient in many countries in the food field. Protein and polysaccharide blend coatings or films can improve their functional properties through intermolecular interactions. Maria et al. [18] prepared nanocomplexes based on egg white protein nanoparticles and bioactive compounds and extended the shelf life of bread to seven days with antifungal edible coatings. Zhou et al. [19] prepared a hyaluronic acid (HA)-based multifunctional active coating for fruit preservation and found that the coating possessed excellent antioxidant, antimicrobial, and slow-release properties. Sawsan A. et al. [20] investigated an HA-based edible polysaccharide–protein coating for strawberry preservation and showed that the incorporation of HA significantly enhanced the antioxidant properties. The above indicates that EWP and HA can be used as edible films or coatings matrices, and there have been no reports on coatings or films with composite EWP and HA.

Currently, there are few studies on the incorporation of essential oil NEs into edible coatings or films, and there are no studies on egg white protein/hyaluronic acid coatings containing thymol NEs. Therefore, in this study, thymol NEs were prepared by ultrasonic emulsification to compound egg white protein and hyaluronic acid in order to prepare a composite edible coating. The poor performance of coatings prepared using EWP and HA alone limits their application. The addition of HA compensates for deficiencies such as the brittleness of the pure EWP coating and improves the adhesion of the coating to the food surface. The physicochemical and antibacterial properties of the coatings were improved by the addition of thymol NEs and used as food active packaging.

## 2. Materials and Methods

### 2.1. Materials

Thymol (THY, 99% purity) was obtained from Yuanye Biotechnology Co., Ltd. (Shanghai, China). Egg white protein powder (EWP) was obtained from Zhongnong Xinghe Biological Technology Co. Ltd. (Harbin, China). Hyaluronic acid (HA, molecular weight 800–1500 kDa) was obtained from Macklin Reagent Co., Ltd. (Shanghai, China). Surfactant Tween 80 (T-80) and co-surfactant propylene glycol (PG) were obtained from Fuyu Fine Chemical Co. (Tianjin, China). Corn oil was obtained from Biyoute Supermarket (Harbin, China). *Escherichia coli* and *Staphylococcus aureus* strains were obtained from Guangdong Microbial Strain Preservation Center. The water used for sample preparation was deionized water.

### 2.2. Preparation of Thymol Nanoemulsions

For the preparation of oil-in-water nanoemulsion, corn oil containing thymol was used as the dispersed phase and deionized water as the continuous phase. It is known from previous studies that the surfactant to oil phase (OP) ratio SOR = 1 is most suitable [14]. The total oil phase (10%, *w*/*w*) in this experiment consisted of different mass fractions of thymol (from 0–50%, *w*/*w*) and corn oil as a carrier oil. The oil phase and Tween 80 (10%, *w*/*w*) were firstly mixed by a magnetic stirrer (500 rpm) for 30 min, followed by the addition of PG and deionized water (80%, *w*/*w*) to prepare a crude emulsion (THYCE), which was processed by using a high-speed shear at 10,000 rpm for 2 min [21]. Subsequently, ultrasonic homogenization (USF) was used to convert the crude emulsion into nanoemulsion (NE). A titanium probe with a diameter of 6 mm was placed inside the sample at a depth of 25 mm and the sonication process (400 W, 20 kHz) was carried out for 10 min at 20 °C. The temperature was controlled using an ice water bath so as to not exceed 20 °C.

### 2.3. Characterization of Thymol Nanoemulsion

#### 2.3.1. Particle Size, Polydispersion Index, and Zeta Potential

The mean particle size, polydispersity index (PDI), and zeta potential of thymol nanoemulsion (THYNE) were determined using a Zetasizer Nano ZS-90 particle size potential analyzer (Malvern, UK) at 25 °C based on the dynamic light scattering (DLS) technique with a scattering angle of 90°. THYNE was diluted 100 times with deionized water before testing to avoid multiple scattering effects [22].

#### 2.3.2. Physical Stability and Storage Stability

The centrifugal acceleration method was used to evaluate the physical stability of THYNE [23]. The appropriate amount of emulsion was diluted 100 times, and its absorbance value was measured at 600 nm by UV–visible spectrophotometer to obtain its absorbance value A. Calculate the turbidity according to the following Formula (1):

(1) T=2.303×A×D/L

where *A* is the absorbance of the sample, *D* is the dilution, and *L* is the path length of the cuvette (cm).

To evaluate the stability of the emulsions, the samples were centrifuged at 10,000 rpm for 10 min at room temperature to observe whether THYNE showed phase separation. After centrifugation, the absorbance value of the emulsion was measured at 600 nm to obtain A0. The centrifugal stability constant Ke (%) was used to express the centrifugal stability of NE, and the smaller the value of Ke, the more stable the emulsion was. The formula was calculated as follows (2):

(2) $$Ke=\left[\left(A-A_{0}\right)/A\right]\times 100$$

where *A* and *A*_0_ are the absorbance of THYNE before and after centrifugation, respectively.

The prepared NEs were stored at 4 °C and the particle size, PDI, and zeta potential of the emulsions were measured at 0, 7, 14, 21, and 28 days to evaluate the storage stability of THYNE.

#### 2.3.3. pH Stability

Freshly prepared THYNE samples were placed in glass tubes, the nanoemulsions were adjusted to different pH values (2–7) using hydrochloric acid and sodium hydroxide and the emulsions were observed for the occurrence of phase separation. The average particle size, PDI and zeta potential of THYNE at different pH levels were then determined.

#### 2.3.4. Microscopic Observation

An appropriate amount of NE was aspirated with a pipette gun and uniformly spread on a dry and clean slide, and the coverslip was covered gently. The distribution of droplets was observed under an optical microscope and the selected interface was photographed with an accompanying camera [24].

### 2.4. Preparation of Composite Coatings of Egg White Protein and Hyaluronic Acid

Firstly, 8.0 g of EWP was weighed in a 100 mL beaker and stirred gently until completely dissolved to obtain 8% (*w*/*v*) egg white protein solution, which was hydrated overnight at 4 °C. Then, 0.4 g of HA was weighed in a 100 mL beaker and stirred at 45 °C for 60 min to obtain 0.4% (*w*/*v*) hyaluronic acid solution. The two solutions were mixed well according to the volume ratio of 1:1, and 30% of glycerol (by weight of EWP and HA) was added and stirred for 30 min to obtain a coating solution containing 4% (*w*/*v*) EWP and 0.2% (*w*/*v*) HA. Finally, THYNE, at different volume fractions (0, 5%, 10%, 20%), was added and stirred for 30 min to obtain the coating solution containing different THYNE contents.

### 2.5. Rheological Properties of Composite Coating Solutions

The rheological properties of coating solutions containing different THYNE contents were determined using a MARS40 modular rotational rheometer (Thermo Fisher Scientific, Waltham, MA, USA). A flat plate with a diameter of 60 mm was selected and the gap was set to 0.3 mm. The samples were placed in the rheometer and equilibrated at 25 °C before analysis. The shear rate was increased from 0.1 s^−1^ to 100.0 s^−1^, and the variation of the apparent viscosity of coating solutions with shear rate was observed [25].

### 2.6. SEM of Composite Coatings

Scanning electron microscopy (SEM) was used to observe the microstructure of the surface and cross-section of coatings containing different THYNE contents (S-3400N SEM, Hitachi, Japan). The coatings were fixed on a specimen holder with the cross-section of the coating facing upwards using double-sided adhesive tape, and then the samples were sputter-plated with gold under vacuum conditions to make them electrically conductive. An accelerating voltage of 5 kV and a magnification of 500 times were used to observe the structural images of the samples [26].

### 2.7. FTIR Characterization of Coatings

The structures of EWP coatings, EWP/HA coatings, and EWP/HA coatings containing THYNE were analyzed using a FTIR spectrometer (Nicolet Magna 4R 560, Nicolet, Madison WI, USA). The samples were proportionally mixed with KBr, ground and pressed, and scanned and recorded in attenuated total reflection (ATR) mode in the wavelength range of 4000 to 500 cm^−1^ and with a resolution of 4 cm^−1^ [27].

### 2.8. Release of THY from Coatings

The coating solutions were dried at 45 °C for 14 h. The dried coatings were fully dispersed into deionized water, and THY was extracted by adding an equal volume of anhydrous ethanol. Then, the supernatant was collected and diluted in ethanol–water solution. The absorbance values were measured at 274 nm using a UV–visible spectrophotometer (UV-2600, Hitachi, Japan). The amount of THY was calculated from the standard absorbance curve and the retention rate of THY was calculated according to the following Equation (3):

(3) $$Retention\;rate\;of\;THY\left(\%\right)=\frac{M_{0}}{M}\times 100$$

where *M*_0_ is the content of THY in coatings and *M* is the amount of THY added to the coatings.

Two standard food simulants were selected: deionized water (for simulating foods with high water content) and 95% (*v*/*v*) aqueous ethanol solution (for simulating foods with high fat content) [28]. The dried coatings were soaked in 100 mL of the food simulant solution with gentle stirring at 25 °C. The supernatant was collected at specific time intervals, with the addition of the same quantity of fresh food simulant solution. The absorbance value of the supernatant was measured at 274 nm using a UV–vis spectrophotometer and the amount of THY released was calculated according to the following Equation (4):

(4) $$Release\;rate\;of\;THY\left(\%\right)=\frac{M_{t}}{M_{0}}\times 100$$

where *M_t_* is the amount of THY released at moment *t* and *M*_0_ is the amount of THY retained in the dried coating.

### 2.9. Antibacterial Activity Evaluation of Coatings

According to Yuan et al. [29], the antibacterial activity of the coating solution against *Staphylococcus aureus* and *Escherichia coli* at 25 °C was determined by the agar diffusion method. About 15 mL of LB agar medium was added to a Petri dish, and, after it was cooled and solidified, 100 uL of bacterial suspension with a concentration of 10^5^ CFU/mL was added to the surface of the medium and spread uniformly. A 10 mm sterile filter paper sheet was soaked in the coating solutions, and after sterilization, the filter paper sheet was spread flat on the agar plate and incubated at 37 °C for 24 h. The diameter of the inhibition zone was observed and measured.

### 2.10. Statistical Analysis

Three parallel determinations were used for all experimental data. Graphs were plotted using Origin 2022 software (Originlab Inc., Northampton, MA, USA). Analysis of variance (ANOVA) and significance tests were performed using SPSS software (version 25, IBM Inc., New York, NY, USA). The level of significance was set at *p* < 0.05.

## 3. Results and Discussion

### 3.1. Effect of Different Oil Phase Compositions on Particle Size, PDI, and Zeta Potential of Nanoemulsions

**Figure 1:**
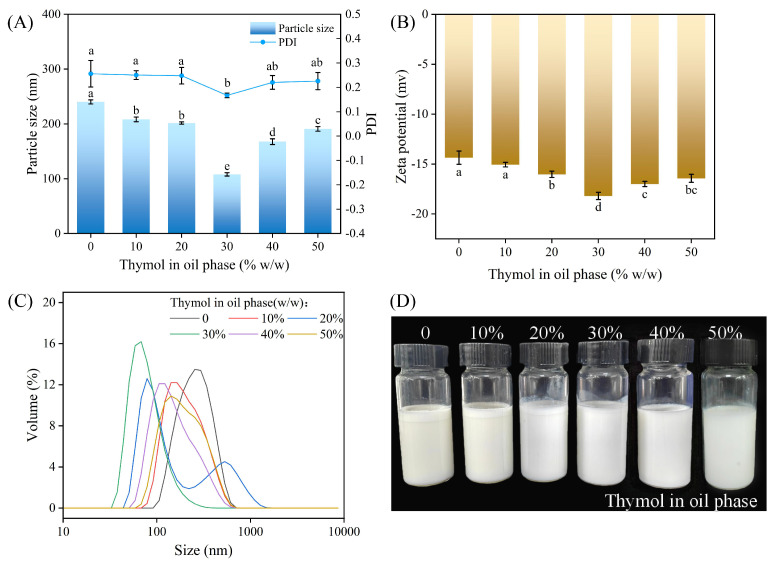
Particle size, PDI, and Zeta potential of THYNE with different oil phase compositions: (**A**) average particle size and PDI; (**B**) Zeta potential; (**C**) particle size distribution map; (**D**) physical image of nanoemulsion after 1 day. Different letters (a–e) indicate significant differences (*p* < 0.05).

The NE system was used with SOR = 1 (10% *w*/*w* total oil (THY + corn oil), 10% *w*/*w* surfactant, 80% *w*/*w* aqueous phase). Corn oil was chosen as the carrier oil because it is rich in long-chain triacylglycerols, which are widely used in food production, and which can be used as a maturation inhibitor of the emulsion, so as to maintain its long-term stability. Tween 80 was chosen as the surfactant because many studies have shown that, as a nonionic surfactant, it is suitable for the preparation of NE and because it stabilizes the emulsion through spatial stabilization [30]. In general, emulsions with smaller particle sizes are more stable, and the smaller the PDI value, the narrower the droplet size distribution, indicating a more homogeneous emulsion [31]. From Figure 1A, the average particle size and PDI decreased gradually as the THY content in the OP increased from 0 to 30%, and when the OP consisted of 30% THY and 70% corn oil, the THYNE particle size reached a minimum value of 107.93 nm and the PDI reached 0.167. These values are similar to those found in the results of Robledo et al. [14]. The decrease in the particle size and the increase in the homogeneity of the emulsions may be attributed to the increase in the content of some amphiphilic fractions of the THY that enhance the surfactant activity [32] and the inhibition of Ostwald ripening (OR) by sufficient concentration of corn oil. When the THY content was greater than 30%, the average particle size and PDI increased subsequently, probably due to oil droplet agglomeration caused by high THY concentration.

The results in Figure 1B show that the prepared NEs all have negative Zeta potentials, which can be attributed to mechanisms such as the ionization of functional groups and the adsorption of ions that occurs because the phenolic hydroxyl groups (-OH) of THY molecules can partially ionize in aqueous solution to produce anions. In addition, the anionic groups on the THY molecule and T80 undergo ionic adsorption at the oil–water interface, generating a negative charge [33]. When the THY content in the oil phase was 30%, the Zeta potential reached −18.2 mV. It is generally accepted that an absolute Zeta potential higher than 30 mV indicates a sufficiently strong electrostatic repulsion between droplets. In contrast, THYNE prepared in this study remained relatively stable at low potentials, which is mainly because the main effect of T80 in preventing agglomeration is steric stabilization rather than electrostatic stabilization [34]. Figure 1C shows that the particle size distribution of the NEs is unimodal, with the same trend variation as that of the average particle size and PDI. The droplet size distribution is narrowest when the THY content is 30%. Figure 1D shows the physical picture of the prepared NEs, which is milky white in color. When the THY content in the OP was 0–20%, there was a slight oil–water separation in the NE, and when the THY content was more than 30%, there was no oil–water separation and it was more stable.

### 3.2. Physical and Storage Stability of Thymol Nanoemulsions

**Figure 2:**
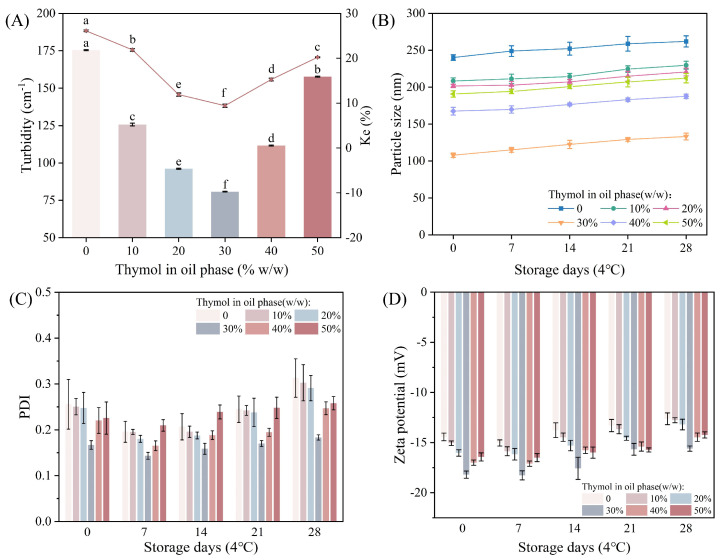
Physical and storage stability of THYNE: (**A**) turbidity and centrifugal stability constant Ke%; (**B**) change in average particle size for 4 weeks of storage; (**C**) change in PDI for 4 weeks of storage; (**D**) change in Zeta potential for 4 weeks of nanostorage. Different letters (a–f) indicate significant differences (*p* < 0.05).

Essential oil emulsions undergo various destabilizing processes such as flocculation, agglomeration, OR maturation, precipitation, etc. during long-term storage [35]. Therefore, when preparing essential oil emulsions, it is important to ensure that the emulsion has good stability. Figure 2A shows that all NEs have low Ke values, not exceeding 30%, and the Ke values are positively correlated with the turbidity of the emulsions before and after centrifugation. This is consistent with the findings of da Silva et al. [33]. The NEs were stable at low Ke values, indicating that there was significant repulsion between the system droplets before and after centrifugation, which could reduce the collision between droplets during storage [23]. No significant phase separation was found for all NEs after centrifugation. When the THY content in OP was 30%, the emulsion had the lowest turbidity and the smallest Ke value, which was better stabilized.

To study the storage stability of the NE, the changes in particle size, PDI, and zeta potential of the emulsions were observed after 4 weeks of storage. Figure 2B shows that the particle size of all emulsions increased slowly, which was attributed to OR, but this change was not significant [33]. After 28 days of storage, the increase in emulsion size did not exceed 40 nm on average, indicating that 4 °C is suitable for emulsion preservation. Figure 2C shows the change in PDI of THYNE during storage. Generally, PDI values less than 0.25 indicate uniform droplets in the emulsion [36]. The PDI values of the NEs during storage were in the range of 0.143 to 0.313, and all of the PDI were found to decrease after 1 week of storage, the PDI increased slowly after 2 weeks of storage, and the emulsions were generally homogeneous. Figure 2D shows the change in Zeta potential of THYNE during storage, and it was found that the potential value of each nanoemulsion decreased to a different extent. Zeta potential can directly affect the stability of the emulsion, and the higher absolute value of the potential means that the emulsion system is more stable. The potential values of the NEs were relatively stable during storage ranging from −12.76 to −18.26 mV.

### 3.3. pH Stability of Thymol Nanoemulsion

**Figure 3:**
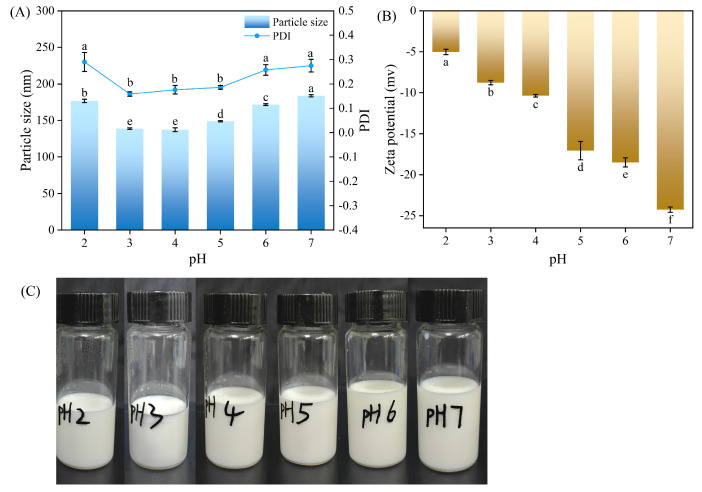
Effect of different pH on THYNE prepared with 30% THY content in the oil phase: (**A**) particle size and PDI; (**B**) Zeta potential; and (**C**) physical appearance. Different letters (a–f) indicate significant differences (*p* < 0.05).

Generally, food has a wide pH range, so the stability of THYNE at different pH was investigated. As shown in Figure 3A, the particle size and PDI of THYNE varied slightly in the pH range of 2–7, but remained low [33]. The particle size ranged from 137.3 to 183.6 nm and the PDI ranged from 0.158 to 0.29. It is noteworthy that in Figure 3B, with the increase of pH, the absolute value of the potential of THYNE becomes higher from −5.02 mV to −24.26 mV, which indicates the enhanced stability of the emulsion system. In Figure 3C, the nanoemulsions showed slight phase separation when the pH was 2–3, and no such phenomenon was observed between pH 4–7, and all the emulsions were milky white in colour. In summary, the THYNE can still maintain high stability under different pH conditions, especially between pH 4–7. Therefore, THYNE in this study has a great potential to be used in low-acid foods by incorporating lipophilic food antimicrobials such as thymol into low-acid food to improve food safety. Because most fruits and vegetables are perishable and their pH range is weakly acidic, we prioritise their use in fruit and vegetable preservation.

### 3.4. Microscopic Images of Thymol Nanoemulsions

**Figure 4:**
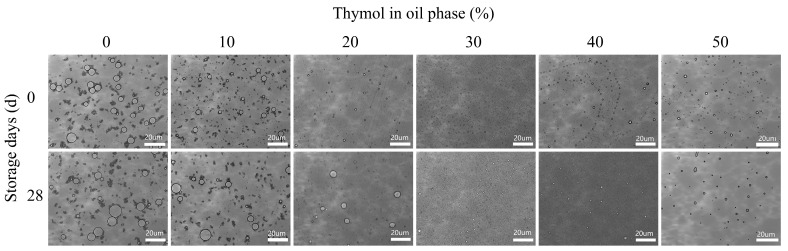
Microscopic images of THYNE prepared with different oil phase compositions on storage days 0 and 28.

Figure 4 shows the microscopic images of the NEs prepared from different oil phase compositions on the 0th and 28th day of storage, and it can be observed that all the emulsion droplets have a spherical structure. From the newly prepared emulsion system, it can be found that the emulsion droplets gradually become smaller and more uniformly distributed with the increase of THY content in the oil phase, but when the THY content exceeds 30%, the emulsion droplets become larger and the uniformity decreases. It was found that the stability of all emulsion systems decreased with the increase of storage time, and the droplets showed agglomeration to become larger and uneven distribution behavior in different degrees [6]. Among them, the highest homogeneity and dispersion of emulsion droplets were maintained at 30% THY content in the oil phase, which is consistent with the previous findings. Therefore, the nanoemulsion with an oil phase composition of 70:30 mass ratio of corn oil to thymol was selected for the subsequent coating solution study.

### 3.5. Rheological Properties of Coating Solutions

**Figure 5:**
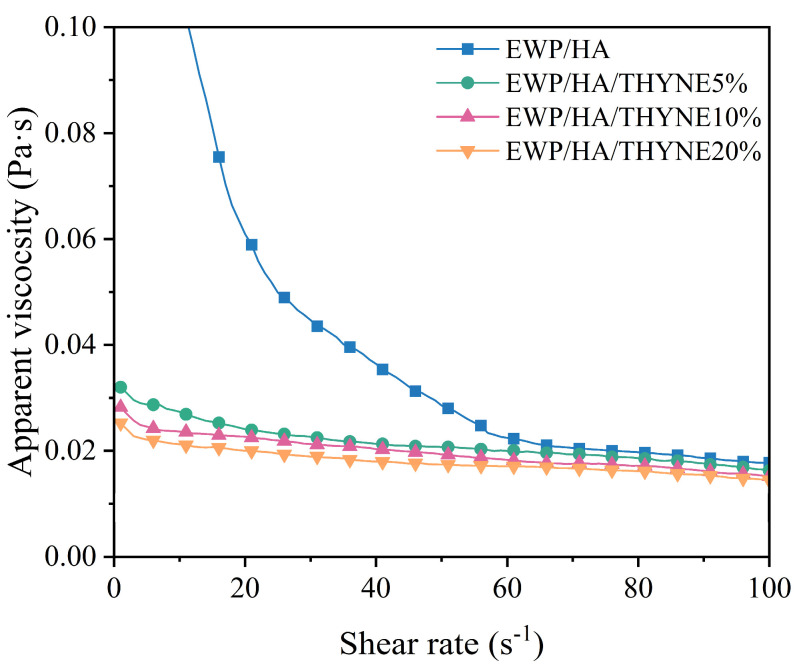
Rheological properties of EWP/HA coating solutions with different THYNE contents.

The rheological properties of coating solutions play a key role in the dispersion, homogeneity, and stability of coatings. Therefore, the study of the rheological properties of the coating solutions can help to understand the microstructure and physicochemical properties of the coating [37]. As can be seen in Figure 5, the apparent viscosity of the coating solution decreases with the increase of shear rate and exhibits shear thinning behavior, which is a characteristic of pseudoplastic fluids (non-Newtonian fluids). This is mainly because the shear external force destroys the molecular network structure formed by EWP and HA in the coating solution, and the physical interactions between polymer molecules are weakened [6]. As the shear rate increases, the speed of molecular re-entanglement to form the network structure is slower than the speed of destroying the network structure, so that the apparent viscosity of the coating soultion is gradually reduced [4]. At the same time, with the increase of THYNE in the coating solution, the apparent viscosity of the coating solution gradually decreases, which may be related to the interference of THYNE with the intermolecular interactions between EWP and HA, and reduces the physical collision between particles in the coating solution. When the content of THYNE in the coating solution is higher, the viscosity decreases, which indicates that the site resistance effect between intermolecular droplets is weakened and the droplets are easily aggregated [4]. Therefore, the addition of THYNE to the coating solution may reduce the structural densification of the coating and make the coating looser.

### 3.6. SEM of Coatings

**Figure 6:**
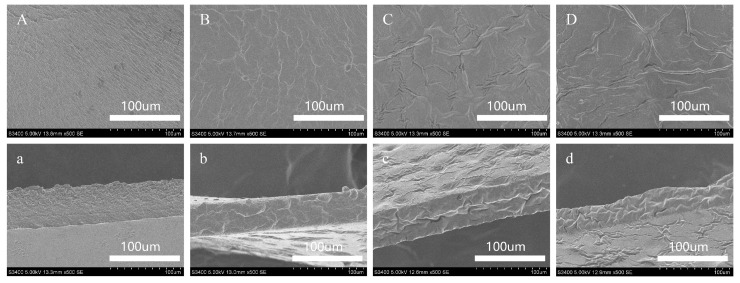
SEM images of surface and cross-section of EWP/HA coatings with different THYNE contents: EWP/HA (**A**,**a**); EWP/HA/THYNE5% (**B**,**b**); EWP/HA/THYNE10% (**C**,**c**); EWP/HA/THYNE/20% (**D**,**d**).

SEM was used to evaluate the micromorphology of the EWP/HA coatings and the distribution of THYNE droplets in the coating matrix to investigate the effects caused by different amounts of added THYNE on the coating properties. Figure 6 shows 500 × SEM surface and cross-section images of EWP/HA coating and EWP/HA coatings containing different THYNE contents. It can be seen that the EWP/HA coatings are relatively smooth and poreless, indicating that EWP and HA are intertwined with each other through intermolecular interactions to form a dense network structure. After the addition of THYNE, THY can be uniformly distributed in the coating solution due to the good water solubility of THYNE and its diffusion in the form of small droplets in the coating solution. However, the partial volatilization of THY during the drying process leads to microporosity and inhomogeneity in the microstructure of the surface and cross-section of the coating. And with the increase of THYNE content, the surface of the coating becomes rough. The similar phenomenon was reported in the study by Zhang et al. [12]. This may be due to the hydrophobicity of the o il droplets in THYNE, which led to the aggregation or migration of emulsion droplets to the top of the coating during the coating drying process, thus increasing the roughness and irregularity of the coating structure [38]. The network structure of the coating also becomes loose, which may be related to the interference of THYNE with the molecular interactions of EWP and HA and the effect of oil droplets in THYNE on the network structure [4]. Due to the strong hydrophobicity of THY, the discontinuity of the network structure of the composite coating increased. Although the microstructure of the coating was changed by different THYNE additions, the overall EWP/HA coating system containing THYNE was more stable. In addition, we observed that the edible coating showed an oily appearance when the THYNE content exceeded 20%, so higher THYNE additions were not investigated.

### 3.7. FTIR Analysis of Coatings

**Figure 7:**
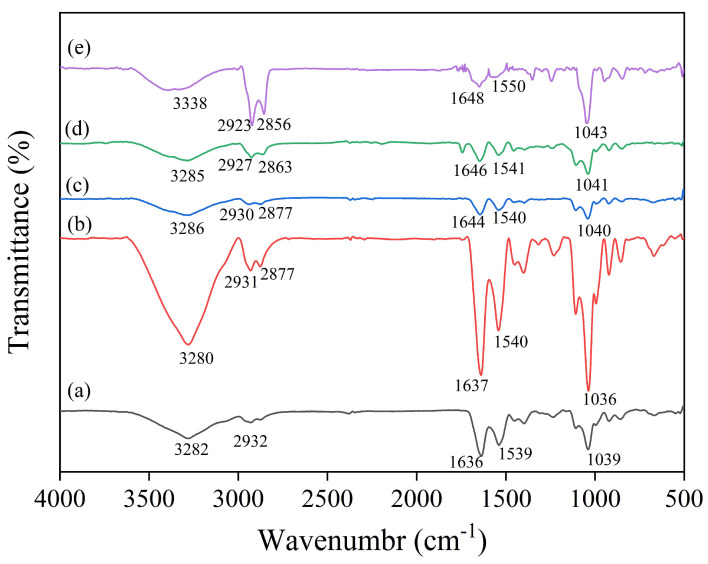
FTIR spectra of EWP/HA/THYNE composite coatings (**a**) EWP; (**b**) EWP/HA; (**c**) EWP/HA/THYNE 5%; (**d**) EWP/HA/THYNE 10%; (**e**) EWP/HA/THNE/20%.

FTIR spectra were used to observe the changes in functional groups and intermolecular interactions of the coating components. The FTIR spectra of the EWP-based coatings prepared with different components are shown in Figure 7. The spectra of the EWP coatings showed characteristic peaks at 3282 cm^−1^ and 2931 cm^−1^ associated with O-H and C-H stretching vibrations, respectively [39]. The peaks at 1636 cm^−1^ and 1539 cm^−1^ are attributed to the C=O stretching vibration (Amide I), the N-H bending vibration and the C-N stretching vibration (Amide II). The peak at 1039 cm^−1^ can be attributed to the stretching vibration of C-O-C [20]. In the spectra of EWP/HA, due to the presence of a large number of hydroxyl and carboxyl groups in HA, the presence of HA shifted the peak of O-H to 3280 cm^−1^, and the peak at 2877 cm^−1^ is due to the stretching vibration of C-H. The peaks at 1637 cm^−1^ and 1540 cm^−1^ are attributed to the C=O and N-H stretching vibrations due to proteins and polysaccharides, and the peak at 1036 cm^−1^ is shifted by the vibration of C-O-C. These changes indicate the presence of hydrogen bonding and electrostatic interactions between EWP and HA molecules [39].

Upon addition of THYNE, the coatings showed similar major peaks but with different intensities of the peaks. With the increase of THYNE, the free O-H stretching vibration on thymol shifted the absorption peak to 3286 cm^−1^, proving the existence of hydrogen bonding interaction between THYNE and the polymer [40]. Meanwhile, the intensity of the peaks at 2931 cm^−1^ and 2877 cm^−1^ increased with the increase of THYNE and the absorption peaks were shifted to the lower wavelengths, which was related to the C-H stretching vibrations of methyl and isopropyl groups on the phenol ring. The same results were found by Karimi-Khorrami et al. [8]. The C=O stretching vibration peak shifted to 1644 cm^−1^ and the C-O-C vibration peak shifted to 1040 cm^−1^, which was attributed to the molecular interactions between THYNE and EWP/HA as well as the formation of stable hydrogen bonds [41]. There was no significant difference between the FTIR spectra of added and unadded THYNE, indicating that no new functional groups were created, and these subtle differences were mainly related to the hydrogen bonding interactions induced by the addition of THYNE and the changes in its orientation structure.

### 3.8. Release Properties of THY from Coatings

**Figure 8:**
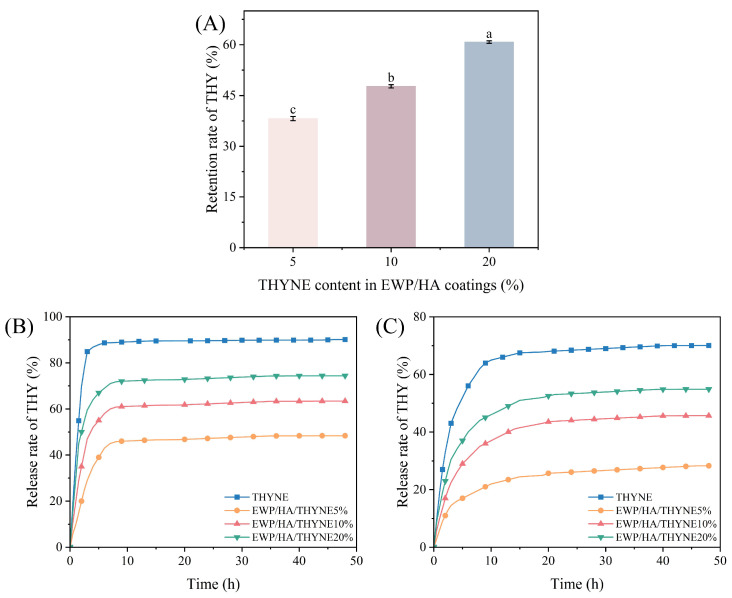
Retention of THY in dried coatings (**A**), release rate of THY from EWP/HA/THYNE coatings in 95% ethanol–water solution (**B**) and deionized water (**C**). Different letters (a–c) indicate significant differences (*p* < 0.05).

The binding of egg white protein and hyaluronic acid macromolecules to thymol is mainly through intermolecular interaction forces, where the polymers are entangled with each other to form a network structure so that thymol molecules are retained in the polymer matrix. However, THY tends to volatilize during the drying process of the coating solution, so the retention of THY in the bacteriostatic coating was explored. Figure 8A shows the retention of THY in coatings with different THYNE contents. The retention of THY increased from 38.20% to 60.81% with increasing THYNE content in the EWP/HA coating. This may be related to the enhanced interaction of THY with the coating matrix due to high THYNE content [42]. Generally, THY was well stabilized in the coating, which was beneficial in maintaining the antimicrobial properties of the coating. Figure 8B,C show the release rate of THYNE and coatings with different THYNE contents in deionized water and 95% ethanol–water solutions of THY. It can be found that the release rate of THY in 95% ethanol–water solution was greater than in deionized water and increased with time. This is due to the high solubility of THY in polar solvents such as ethanol, indicating that the food simulant matrix greatly influences the release of the active substance [43]. Our findings are in agreement with the report of Karimi-Khorrami et al. [8] with sodium alginate films containing nanostructured lipid carriers and nanoemulsions. In 95% ethanol solution, THY was released rapidly for the first 5 h and then slowly to reach equilibrium, while in deionized water THY was released for more than 20 h to reach equilibrium, which is favorable for application in food preservation packaging. When THYNE content in the coating increases, the THY release rate increases, which is because higher THYNE content affects the microstructure and properties of the coating. This is in agreement with the report of Lou et al. [4].

### 3.9. Antibacterial Activity of Coatings

**Figure 9:**
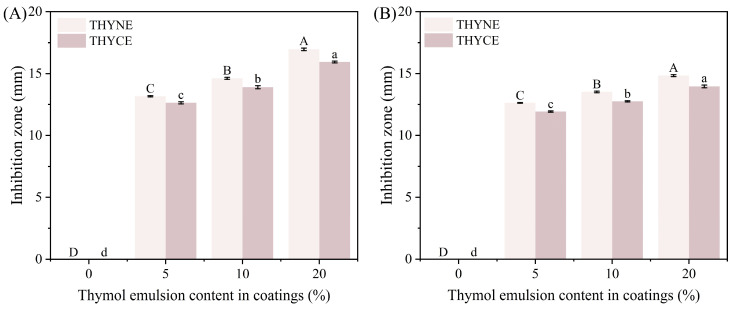
Antibacterial activity of coating solutions with different THY emulsions content against *Staphylococcus aureus* (**A**) and *Escherichia coli* (**B**). Different letters indicate significant differences.

Thymol, the main component of thyme essential oil, has a strong inhibitory effect on both Gram-positive and Gram-negative bacteria. Its antimicrobial mechanism is mainly that thymol can bind to intracellular targets and membrane proteins, causing changes in permeability inside and outside the cell membrane and leakage of cellular contents, leading to cell death, and achieving the effect of bacterial inhibition [44]. In order to investigate the effects caused by the incorporation of different contents of thymol nanoemulsion (THYNE) and thymol crude emulsion (THYCE) on the antimicrobial properties of the EWP/HA coatings, the antimicrobial effect of the composite coatings against *S. aureus* and *E. coli* was assessed by observing the size of the inhibition zone. In Figure 9, it can be seen that the EWP/HA coating has no antimicrobial activity, and with the increase of THYNE content, the antimicrobial activity of the coatings containing THYNE was significantly enhanced, and the inhibitory activity against *S. aureus* was stronger than that against *E. coli*. The same trend was observed for THYCE. This is due to the fact that as a Gram-positive bacterium *S. aureus* has a simpler cell wall structure, thicker peptidoglycan, and no outer membrane, which makes it easy for the active substance to penetrate. In contrast, the cell wall of Gram-negative *E. coli* has a complex structure, hydrophilic membrane, and lipopolysaccharide molecules, which become an obstacle to hydrophobic active substances and reduce their penetration into the cell membrane. This is consistent with the stronger antibacterial activity of THY against Gram-positive bacteria reported by Venkata Giridhar et al. [45]. In addition, the coatings containing THYNE showed significantly a larger inhibition zone against both microorganisms than those containing THYCE, indicating that converting the crude emulsion into nanoemulsion is beneficial to improve the inhibitory activity of the coatings. We find agreement with Zhang et al. in their study of incorporating thyme essential oil nanoemulsions into chitosan composite films [12]. This is because the THYNE droplets emulsified by ultrasonic emulsification have good water solubility and nanoscale size, the smaller size can increase the specific surface area of THY particles, thus expanding the contact area between particles and bacteria [46]. This is one of the reasons for the good antimicrobial performance of the THYNE-containing coating solution in this study.

## 4. Conclusions

In this study, THYNE was successfully prepared by ultrasonic emulsification with a minimum particle size of 107.93 nm, a PDI of 0.167, and a zeta potential of −18.2 mV, which has good physicochemical stability. It can be applied as a delivery lipophilic antimicrobial system in low-acidic foods with pH between 4–7 to improve microbiological safety. The addition of THYNE to the EWP/HA coatings resulted in a gradual decrease in the apparent viscosity of the coating solution and the microstructure of the coatings became sparser. FTIR indicated the presence of hydrogen bonding interactions between THYNE and EWP/HA, which may affect the physicochemical properties of the coatings. The release of THY in the composite coatings was affected by the THYNE content and the substrate of the food simulants. The THYNE-containing coatings showed stronger antimicrobial activity than the THYCE-containing coatings, with better inhibition of both *Staphylococcus aureus* and *Escherichia coli*. This study demonstrated that the incorporation of THYNE into EWP/HA coatings can be used as a low-acidic food packaging material to extend the shelf life of food, such as in fruits and vegetables.

## Data Availability

The original contributions presented in this study are included in the article. Further inquiries can be directed to the corresponding author.
